# Annotations

**Published:** 1892-01-30

**Authors:** 


### Annotations.

**The Risks of Funerals.**

All of us desire to bury our dead decently and with affectionate solemnity. As civilization advances there is no diminution in the strength of this universal human instinct, but rather an increase. There is also in most people a feeling, entirely worthy, that too much anxious care for oneself is like robbing the dead of the self-abandoning solicitude which we ought to experience. But the living do not live for themselves only; they are fathers and mothers of families whose responsibilities can be discharged by no one so well as by themselves. They are sons and daughters; or men of business; or clergymen, lawyers, and doctors. Duty thefore claims its rights as well as feeling. Some of us may not value our own lives very much, but those lives have a value for our children, our parishioners, our patients, or those to whom we may be under business obligations. We owe it to others, even more than to ourselves, to do all that is right and reasonable for the preservation of our health and life. The risks of funerals as ordinarily conducted are undoubted and very great. If accurate statistics could be compiled we should all be startled by the number of previously healthy persons who have taken chills at the grave-side which have ultimately proved fatal. The numbers would have to be reckoned by hundreds, probably by thousands, every year in England alone. Clergymen, especially, are frequent victims, and even when the chills they contract do not prove fatal immediately, there often results a chronic bronchitis, an asthma, or other chest affection which makes life's duties much more burdensome than they ought to be, and frequently ends in premature death. Something will have to be done. A race like our own that always moves in response to the conclusions of its practical intelligence only requires to have the dangers convincingly brought home to its consciousness. What single fact can be more convincing than the recent mournful and untimely death of the Duke of Clarence and Avondale? It is but too evident that if the Prince had not attended a funeral the week before his death he might now have been alive and well. Many suggestions for changes in the direction of safety have been made in the daily papers. One of the best is that offered by the Rev. J. W. Lewis, of Mountsorrel Vicarage. "I always," says Mr. Lewis, "advise the families to come to the parish church in preference to the cemetery chapel, and always say the prayers in the church or chapel immediately after the lesson. This plan leaves only the words of commital to the ground, and the Lord's Prayer to be said at the grave, which take only about two minutes." If Mr. Lewis' suggestions were universally carried out and it were also made *de rigueur* in high places to stand covered at the graveside during the funeral ceremony many valuable lives would be saved, and no fitting solemnity would be left unobserved.

**What next, after Influenza?**

In his *History of Epidemics in Britain*, Dr. Charles Creighton attaches a great deal of importance to certain facts which are often overlooked, but which constitute what deserves to be called a supplementary law of infection. "There are pestilent infections," says Dr. Creighton, "which do not come readily under the law of poor, uncleanly, and negligent living, in the ordinary sense of the words; and there are some communicable diseases which directly contradict the principle that infection falls upon those who engender it by their mode of life. Unwholesome conditions of living may be trusted to engender disease, but it does not follow that the infection so engendered will fall upon those who lead the unwholesome lives; sometimes it falls upon the class who are farthest removed from them in social circumstances or domestic habits, or who are widely separated from them in racial characters. This principle I believe to be not only a necessary complement to the more obvious rule, but to be itself one of wide application." This general statement the author proves by citing the case of the Black Assizes of the sixteenth century, when it was not the criminals who had occupied the pestilent prisons that were most virulently attacked, but the lawyers, the judges, and the audiences in the courts. Similarly, there seems to be no doubt that yellow fever originated in the pestilent conditions of the slave trade, although the slaves themselves seem to have been, to a large extent, "immune" against it. We are witnessing facts of a like kind in connection with influenza. Whatever may be the real *fons et origo* of the disease, it is certain that the depressing vital conditions under which a majority of our population live, distinctly tend to encourage its spread; and yet we find that in what may be called its "Back-wash" it draws in men and women of all ranks and classes, many of whom live under the healthiest environment which a scientific civilisation can create. The obvious conclusion is that the rich and the prosperous, as well for their own sakes as for the sakes of their poorer neighbours, should make it an important part of their business to bring up the whole sanitary conditions of town and country to a state of reasonable healthfulness. The influenza has in earlier times been the precursor of forms of pestilence much more deadly than itself; it may prove to be so again. If we are, as a people, really intelligent and capable, we shall set ourselves to prepare adequate sanitary defences before a more dangerous enemy than influenza approaches any nearer to our shores.

**Doctors and Lawyers.**

The discussion concerning Legal Procedure which was commenced in the *Times* some weeks ago, and which still lingers on in the form of stray letters to other daily and weekly newspapers, failed to deal with one question that seriously affects the public and the medical profession. The way in which matters purely medical are discussed and decided upon in law courts is a disgrace to lawyers and a scandal to civilisation. It is just as proper to set lawyers to judge medical questions as it would be to ask a butcher the best way of preserving the life of a baby. Doctors themselves are really the parties who are least concerned in the matter. They attend at law courts and give their evidence; they submit to cross-examination and take their fees; and there, so far as they are concerned, the matter usually ends. They may have had to endure with more or less patience a good deal of the natural rudeness of the Bar; and they will almost certainly have been amused and astonished at the fearful and wonderful mistakes which counsel have made in their confident attempts to deal with things they have known nothing at all about. All this has been endured with the best grace at command and forgotten in a few days. But what of the parties to the medical "cause" which has been submitted to the wisdom of the lawyers and judges? They went into court to get justice; and they might have got justice if the judges and lawyers had any proper understanding of the facts with which they were called upon to deal. But how can any class of men deal critically and justly with a set of facts which they cannot by any possibility really understand? If a judge were ill, and one were to propose to place him in charge of a young medical man who had but just obtained his qualification, he would probably be exceedingly angry, for he would consider, and properly too, that the young man would neither have the knowledge nor the experience for dealing with the case in the wisest and most successful way. And yet that newly qualified young man would have spent five years in the scientific study and actual practical work of medicine. Now mark the difference when lawyers are called upon to deal with much more complicated, and often very much more important medical cases than a judge's illness! They do not spend five years, or five weeks, or five days in the scientific study of the question. They probably very seldom spend more than four or five hours. The result is that they never know anything about the facts of the case as they really are. They only know the words that describe it, the quibbles that can be made out of it, and the weak points of their opponents' arguments. We say with decision that there ought to be medical judges to decide medical cases, and medical judges only. As the public becomes more educated and learns to take a just measure of things as they are, the present system will be swept away with indignation like a mass of dusty and unwholesome cobwebs. The existing state of things is an anachronism, and only fit for a civilisation of the rudest and the most elementary kind.

